# Supplementary material for: Sesquiterpene Lactones with Anti-Inflammatory Activity from the Halophyte Sonchus brachyotus DC
Source: Molecules. 2023 Feb 4;28(4):1518. doi: 10.3390/molecules28041518 (PMC9964369; doi:10.3390/molecules28041518)
Supplement: Supplementary file 1 [file molecules-28-01518-s001.zip › molecules-2183623-supplementary.pdf]

Supporting Information

Sesquiterpene lactones with anti-inflammatory activity from the Halophyte *Sonchus brachyotus* DC

Young-Kyung Lee[1], Hangy Lee[1], Yun Na Kim[2], Jun Kang[3], Eun Ju Jeong[2,*] and Jung-Rae Rho[1,*]

[1] Department of Oceanography, Kunsan National University, Jeonbuk 54150, Korea; leeyk@nnibr.re.kr (Y.-K.L); sofn123@naver.com (H.L)
[2] Department of Plant & Biomaterials Science, Gyeongsang National University, Jinju 52725, Korea; skdbssk@naver.com
[3] Department of Marine Biotechnology, Kunsan National University, Jeonbuk 54150, Korea; kang4861@hanmail.net
* Correspondence: jeong.ej@gnu.ac.kr; Tel.: +82-55-772-3224(E.J.J); jrrho@kunsan.ac.kr; Tel.: +82-63-469-4606 (J.-R.R)

Contents

Figure S1. Fingerprint of the BuOH fraction. ·· 3

Table S1. ^{1}H (500 MHz) and ^{13}C (125 MHz) data for compound **2** in CD_3OD. ·· 4

Figure S2. (A) ^{1}H (500 MHz) and (B) ^{13}C NMR (125 MHz) spectra of compound **1** In CD_3OD. ················ 5

Figure S3. (A) ^{1}H (500 MHz) and (B) ^{13}C NMR (125 MHz) spectra of compound **2** In CD_3OD. 6

Figure S4. COSY spectrum (500 MHz) of compound **2** in CD_3OD. --7

Figure S5. HSQC spectrum (500 MHz) of compound **2** in CD_3OD. --8

Figure S6. HMBC spectrum (500 MHz) of compound **2** in CD_3OD. --9

Figure S7. ROESY spectrum (500 MHz) of compound **2** in CD_3OD. --10

Figure S8. (A) ^{1}H (500 MHz) and (B) ^{13}C NMR (125 MHz) spectra of compound **3** In CD_3OD. ---------------11

Figure S9. (A) ^{1}H (500 MHz) and (B) ^{13}C NMR (125 MHz) spectra of compound **4** In CD_3OD. ---------------12

Figure S10. COSY spectrum (500 MHz) of compound **4** in CD_3OD. --13

Figure S11. HSQC spectrum (500 MHz) of compound **4** in CD_3OD. --14

Figure S12. HMBC spectrum (500 MHz) of compound **4** in CD_3OD. --15

Figure S13. ROESY (500 MHz) spectrum of compound **4** in CD_3OD. --16

Figure S14. (A) ^{1}H (500 MHz) and (B) ^{13}C NMR (125 MHz) spectra of compound **5** In CD_3OD. -------------17

Figure S15. COSY spectrum (500 MHz) of compound **5** in CD_3OD. --18

Figure S16. HSQC spectrum (500 MHz) of compound **5** in CD_3OD. --19

Figure S17. HMBC spectrum (500 MHz) of compound **5** in CD_3OD. --20

Figure S18. ROESY (500 MHz) spectrum of compound **5** in CD_3OD. --21

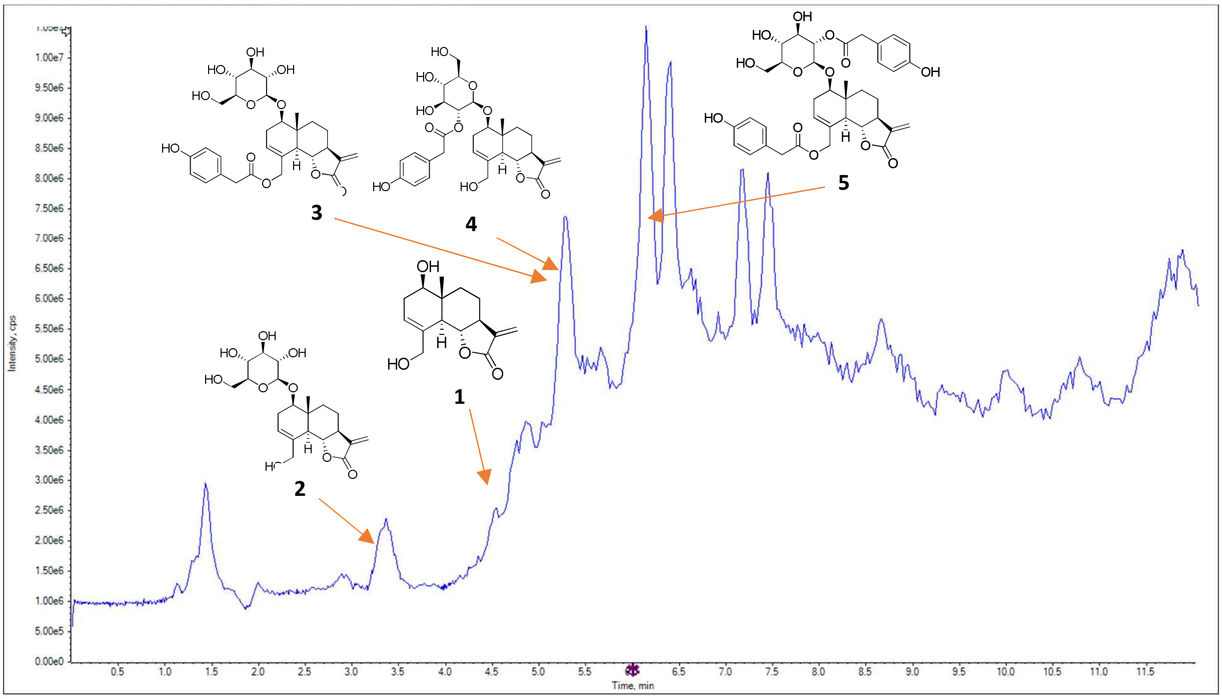

Positive LC-MS chromatography with an AB X500R qTOF mass spectrometer

- Solvents : A - H_2O with 0.1 % formic acid, B - acetonitrile (0–12 min, 10–75% B)
- Column : Synergy Fusion-RP (50 × 2.0 mm)
- Flow rate : 0.3 mL/min

Figure S1. Fingerprint of the BuOH fraction of *S. brachyotus*.

Table S1. ^{1}H (500 MHz) and ^{13}C (125 MHz) data for compound **2** in CD_3OD.
(δ in ppm, *J* values in parenthesis)

no	δ_C	δ_H, mult(*J* Hz)
1	80.9, CH	3.90, dd(9.5, 6.4)
2	30.0, CH_2	α: 2.60, m<br>β: 2.14, m
3	123.1, CH	5.75, br s
4	137.7, C	
5	50.7, CH	2.56, m
6	83.0, CH	4.08, t(11.0)
7	52.0, CH	2.58, m
8	22.2, CH_2	α: 2.09, br d(12.3)<br>β: 1.64, qd(12.3, 3.4)
9	35.6, CH_2	α: 1.47, td(13.7, 1.5)<br>β: 2.15, m
10	41.2, C	
11	140.9, C	
12	172.5, C	
13	117.2, CH_2	5.50, d(3.2); 6.01, d(3.2)
14	12.3, CH_3	0.95, s
15	65.3, CH_2	4.12, d(15.2); 4.16, d(15.2)
1’	101.4, CH	4.35, d(7.8)
2’	75.1, CH	3.16, dd(9.1, 7.8)
3’	78.2, CH	3.34, dd(9.1, 9.5)
4’	71.9, CH	3.26. t(9.5)
5’	78.0, CH	3.24, ddd(9.5, 5.6, 1.2)
6’	63.0, CH_2	3.66, dd(11.7, 5.6)); 3.86, dd(11.7, 1.2)

(A)

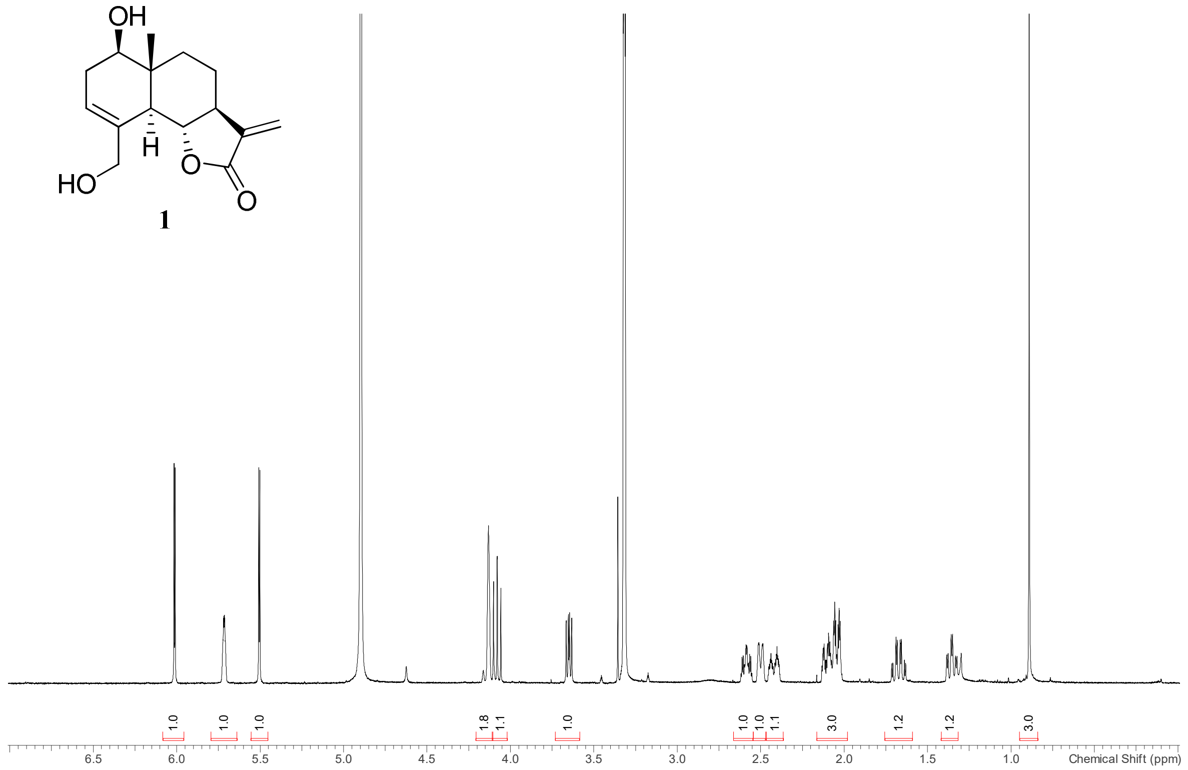

(B)

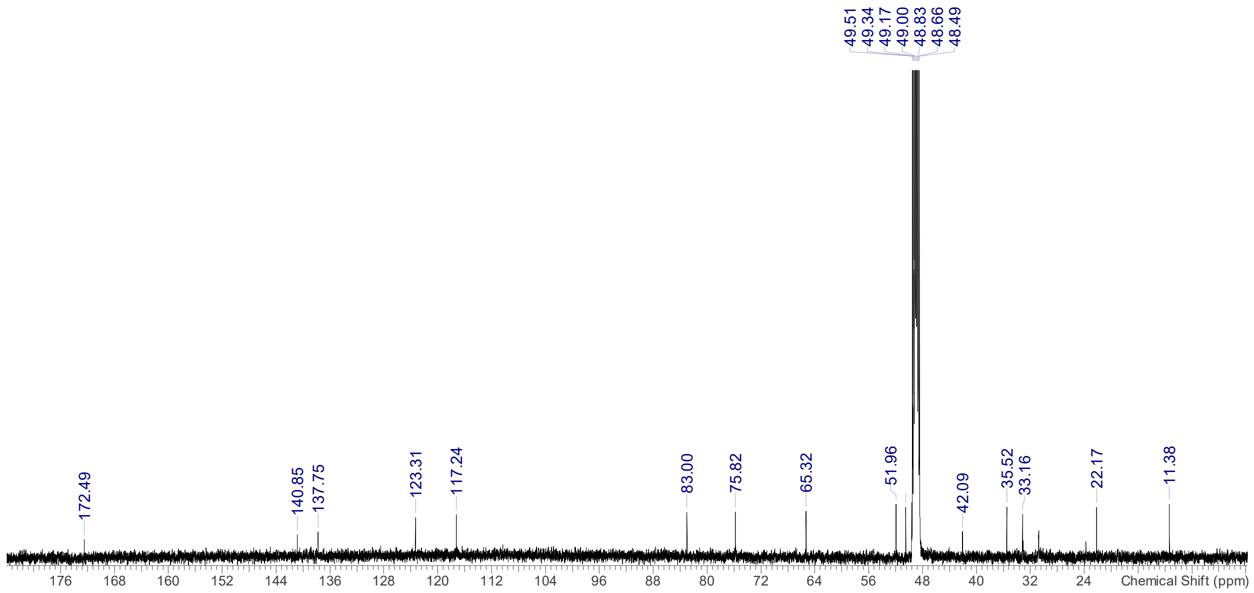

Figure S2. (A) ^{1}H (500 MHz) and (B) ^{13}C NMR (125 MHz) spectra of compound **1** In CD_3OD.

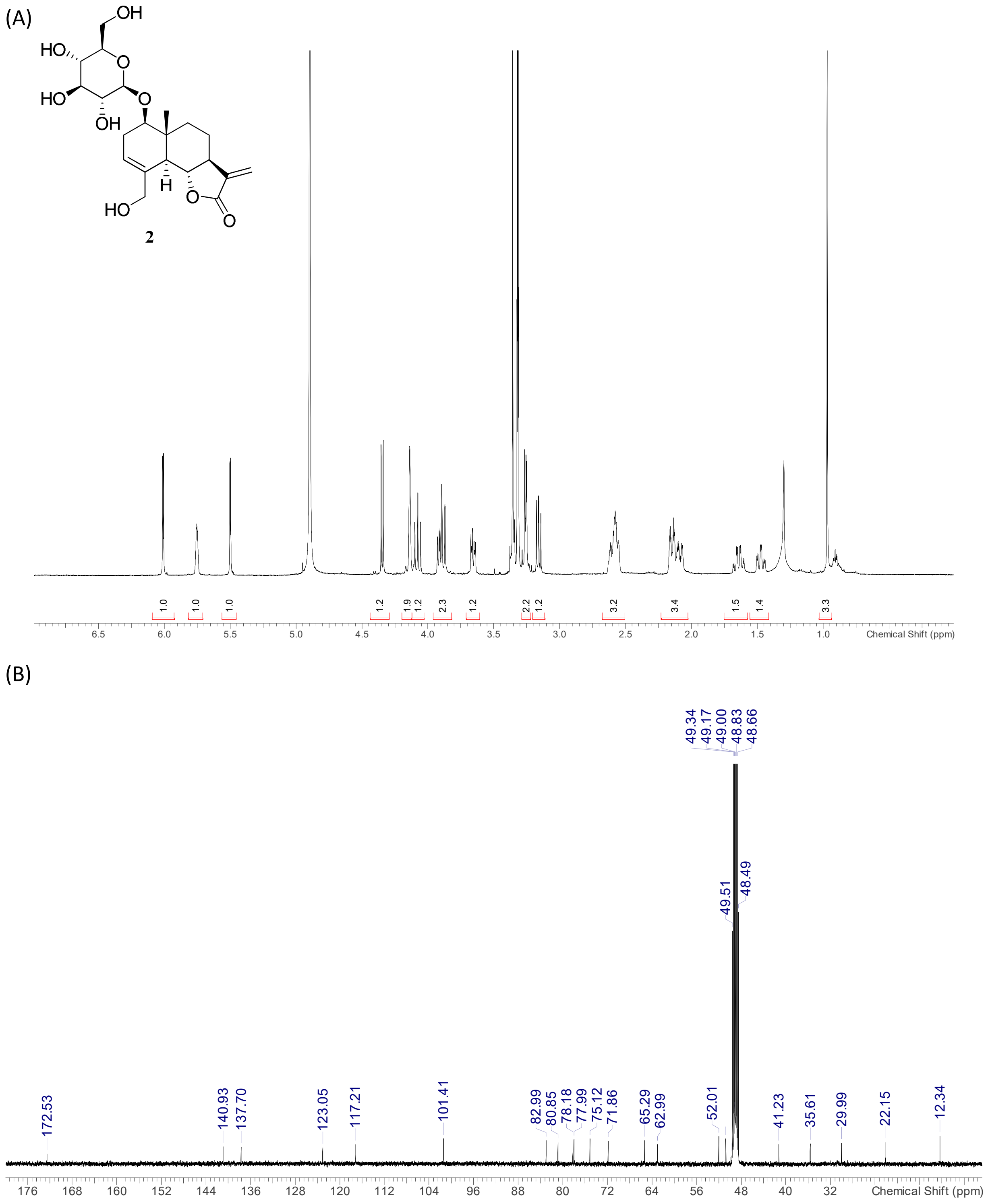

Figure S3. (A) ^{1}H (500 MHz) and (B) ^{13}C NMR (125 MHz) spectra of compound **2** In CD_3OD.

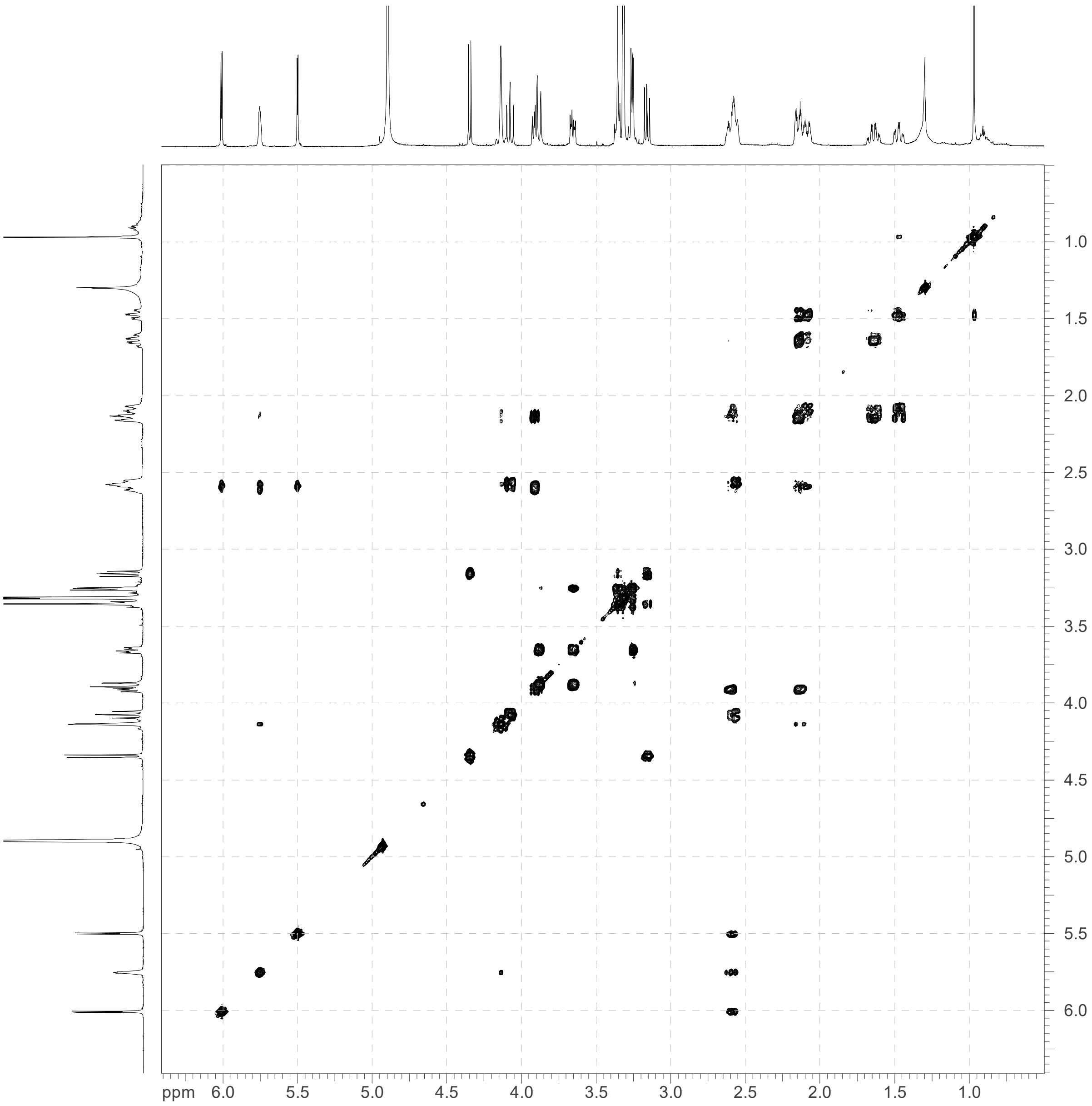

Figure S4. COSY spectrum (500 MHz) of compound **2** in CD_3OD.

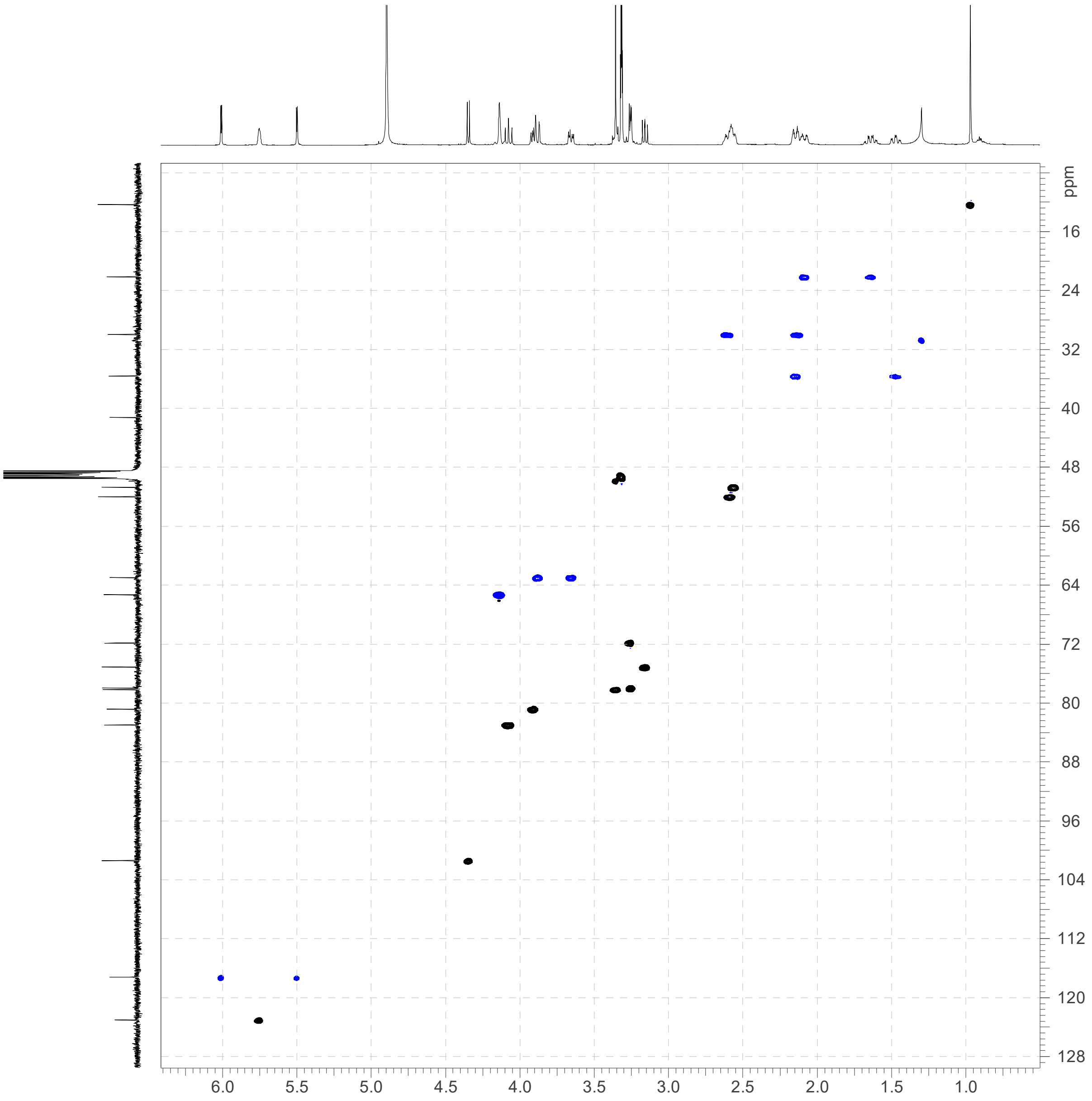

(black: CH/CH_3; blue: CH_2)

Figure S5. HSQC spectrum (500 MHz) of compound **2** in CD_3OD.

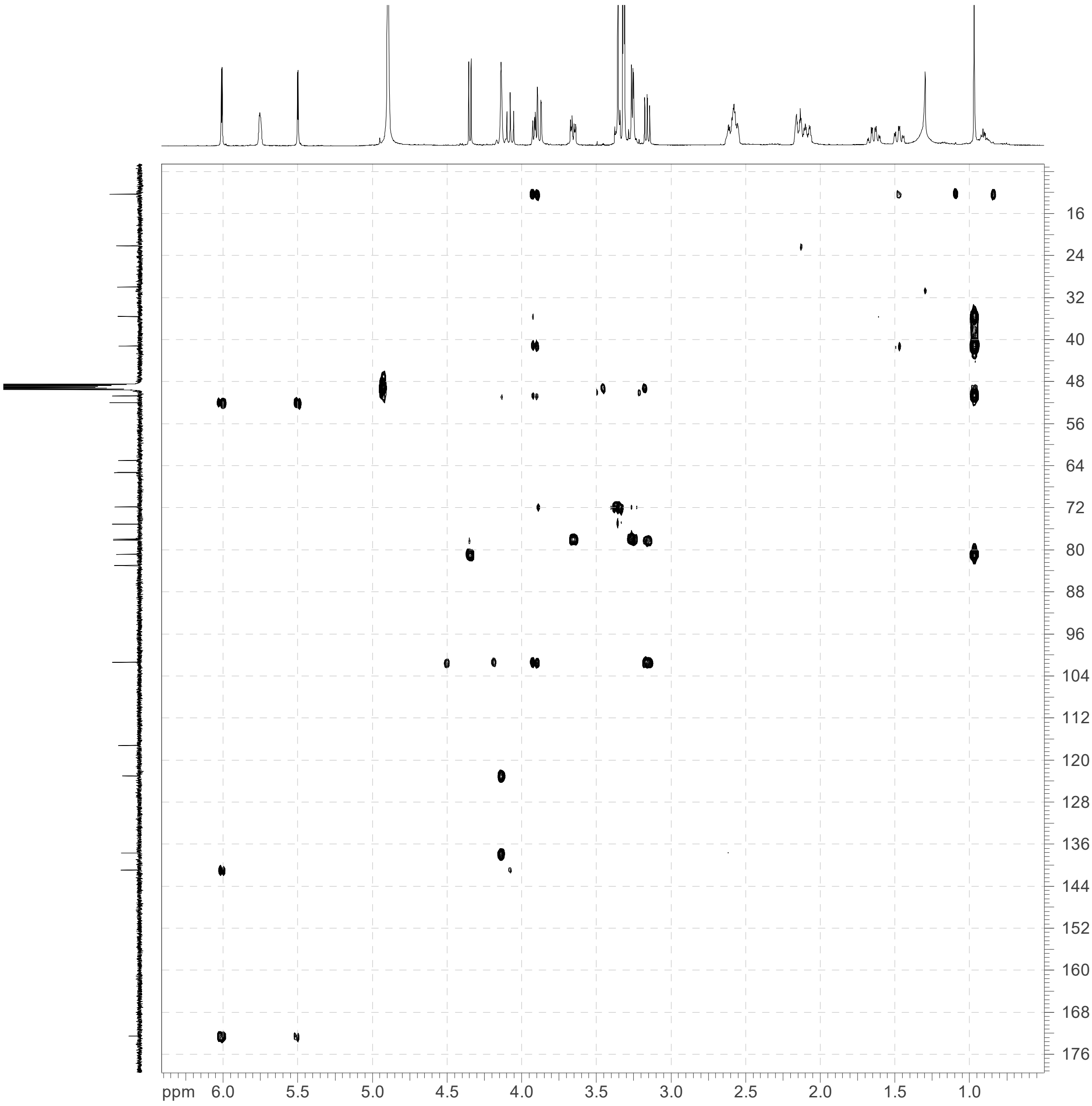

Figure S6. HMBC spectrum (500 MHz) of compound **2** in CD_3OD.

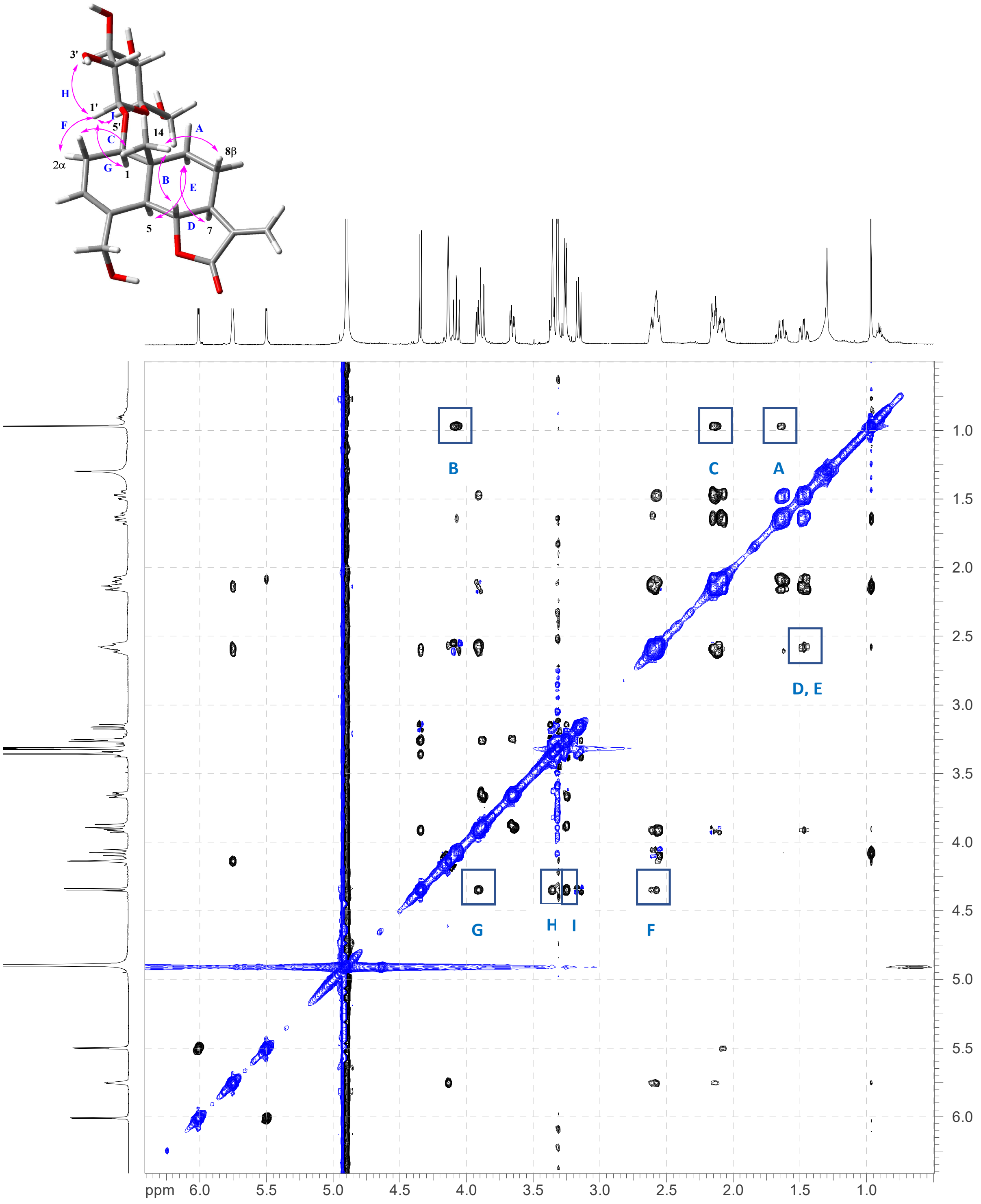

Figure S7. ROESY spectrum (500 MHz) of compound **2** in CD_3OD.

(A)

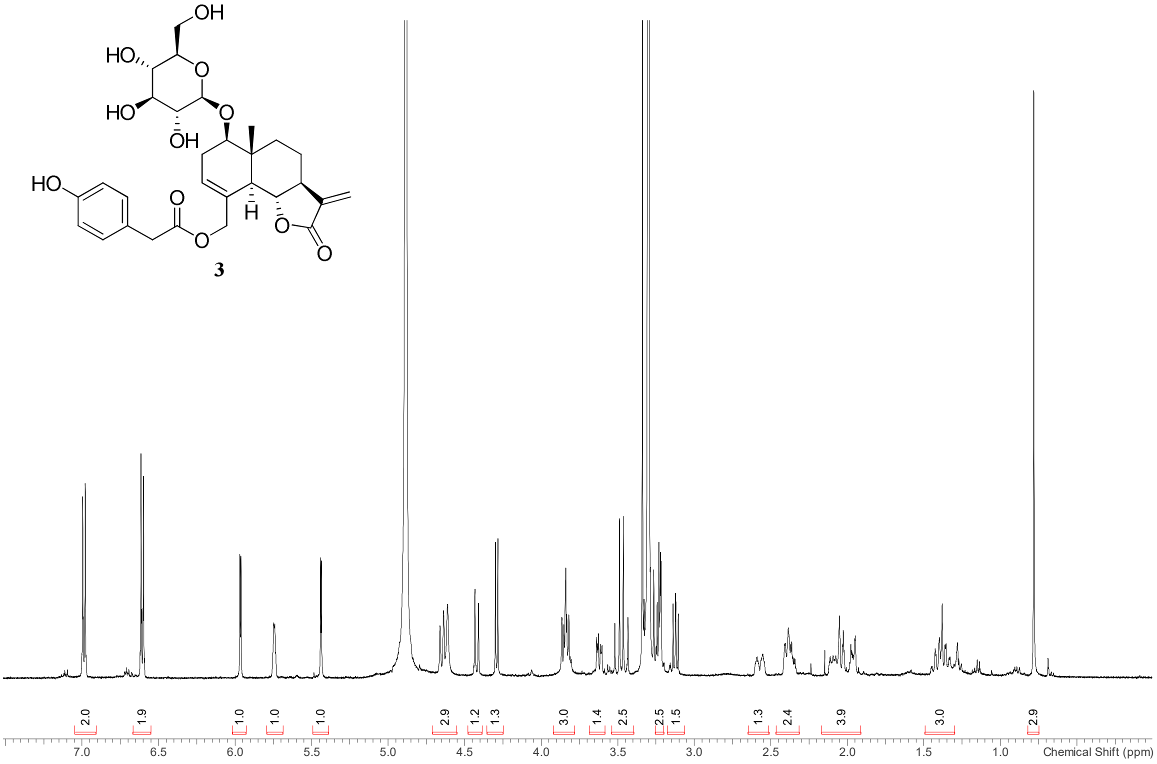

(B)

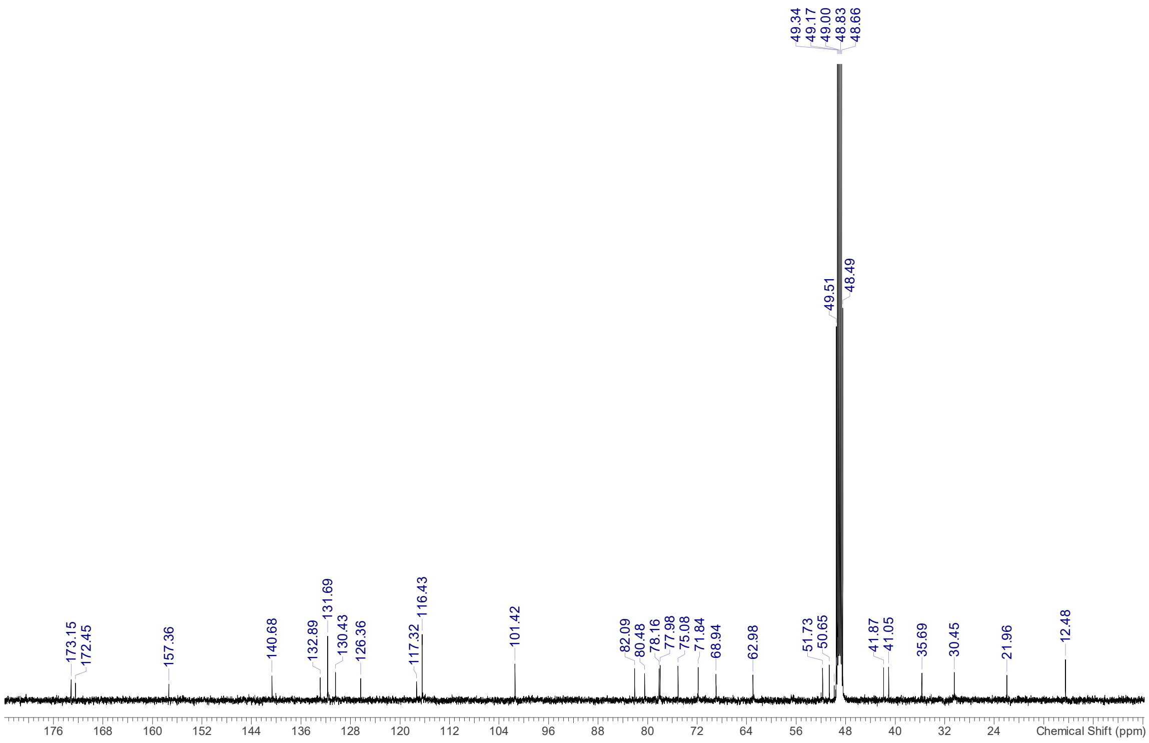

Figure S8. (A) ^{1}H (500 MHz) and (B) ^{13}C NMR (125 MHz) spectra of compound **3** In CD_3OD.

(A)

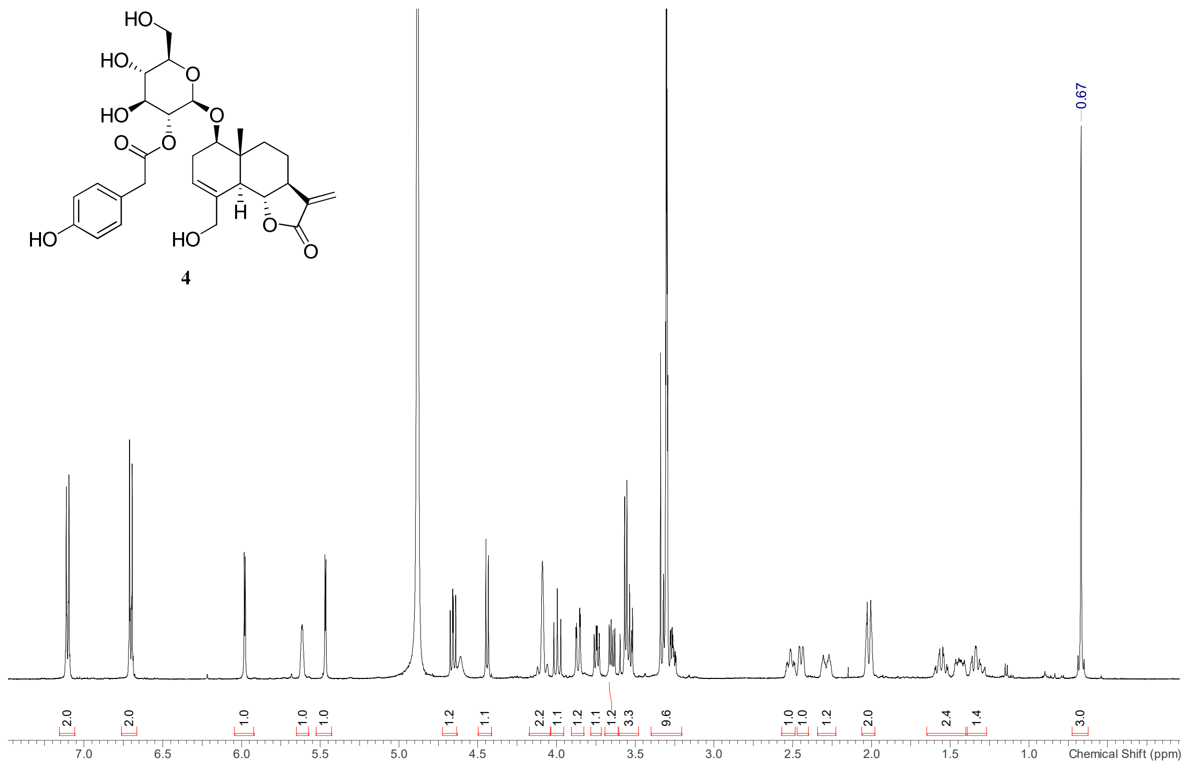

(B)

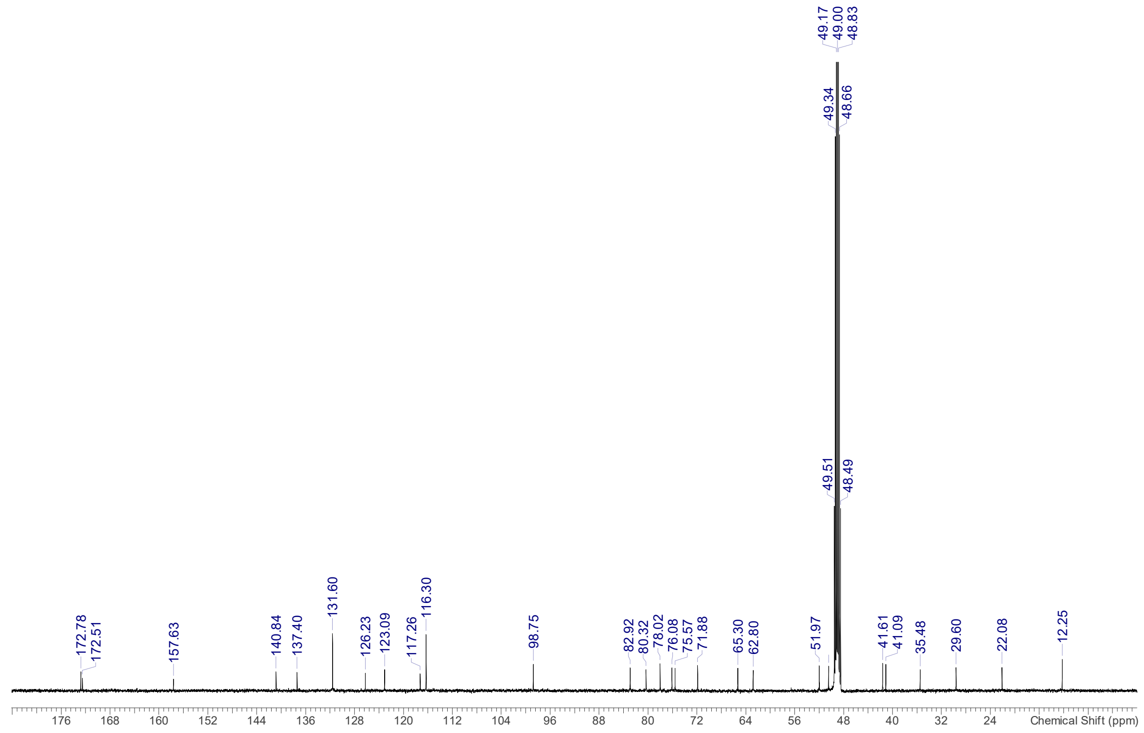

Figure S9. (A) ^{1}H (500 MHz) and (B) ^{13}C NMR (125 MHz) spectra of compound **4** In CD_3OD.

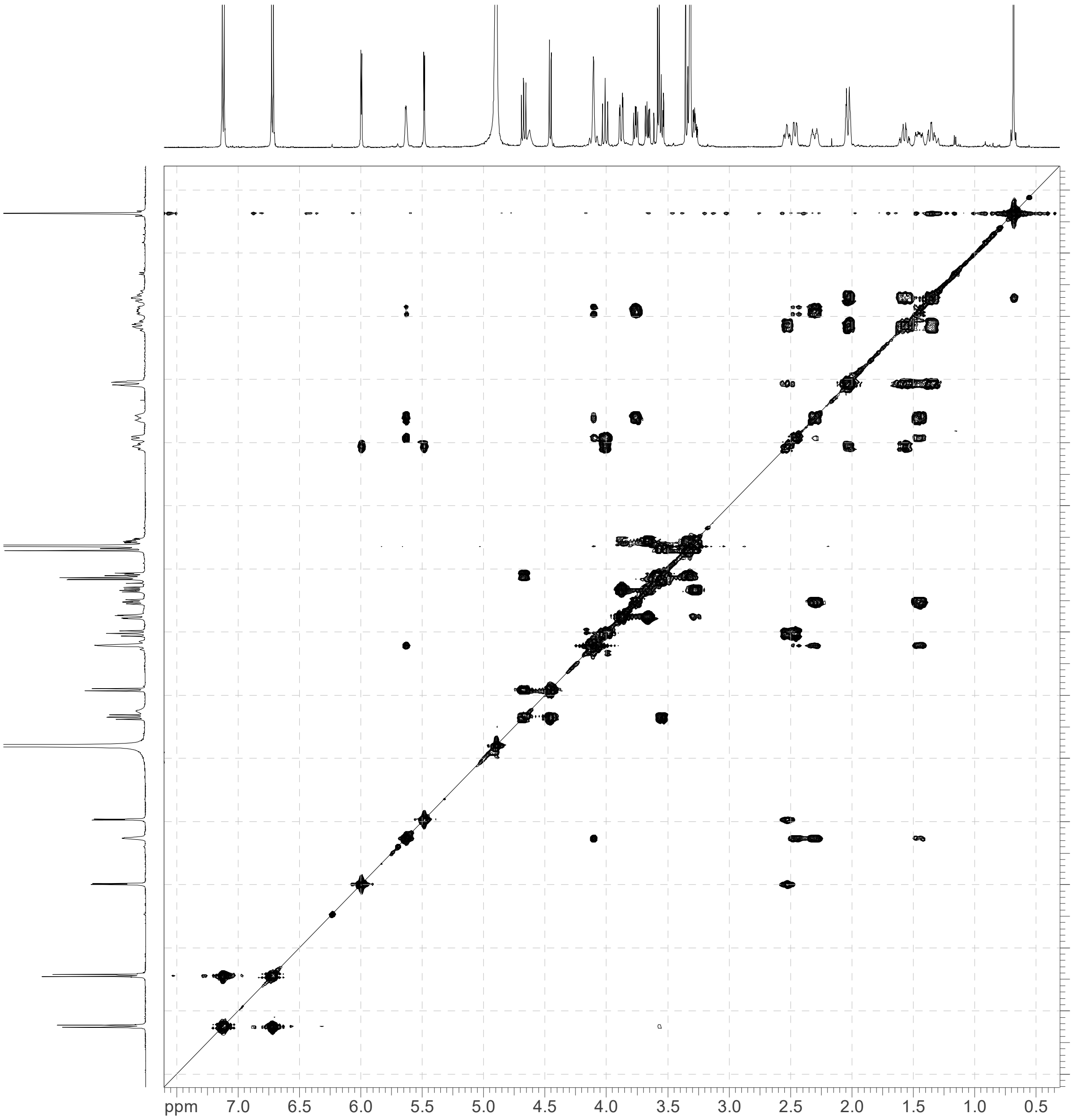

Figure S10. COSY spectrum (500 MHz) of compound **4** in CD_3OD.

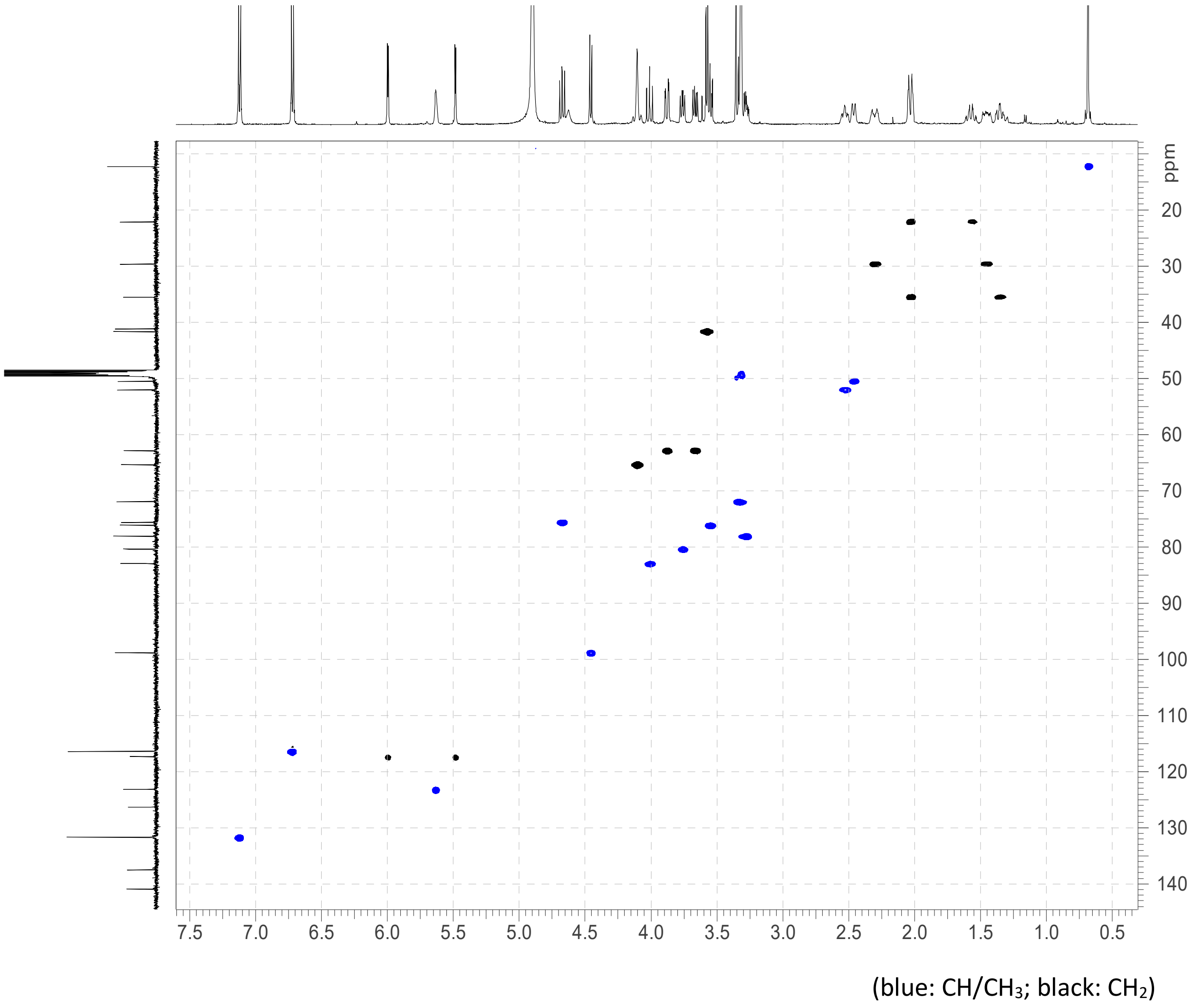

Figure S11. HSQC spectrum (500 MHz) of compound **4** in CD_3OD.

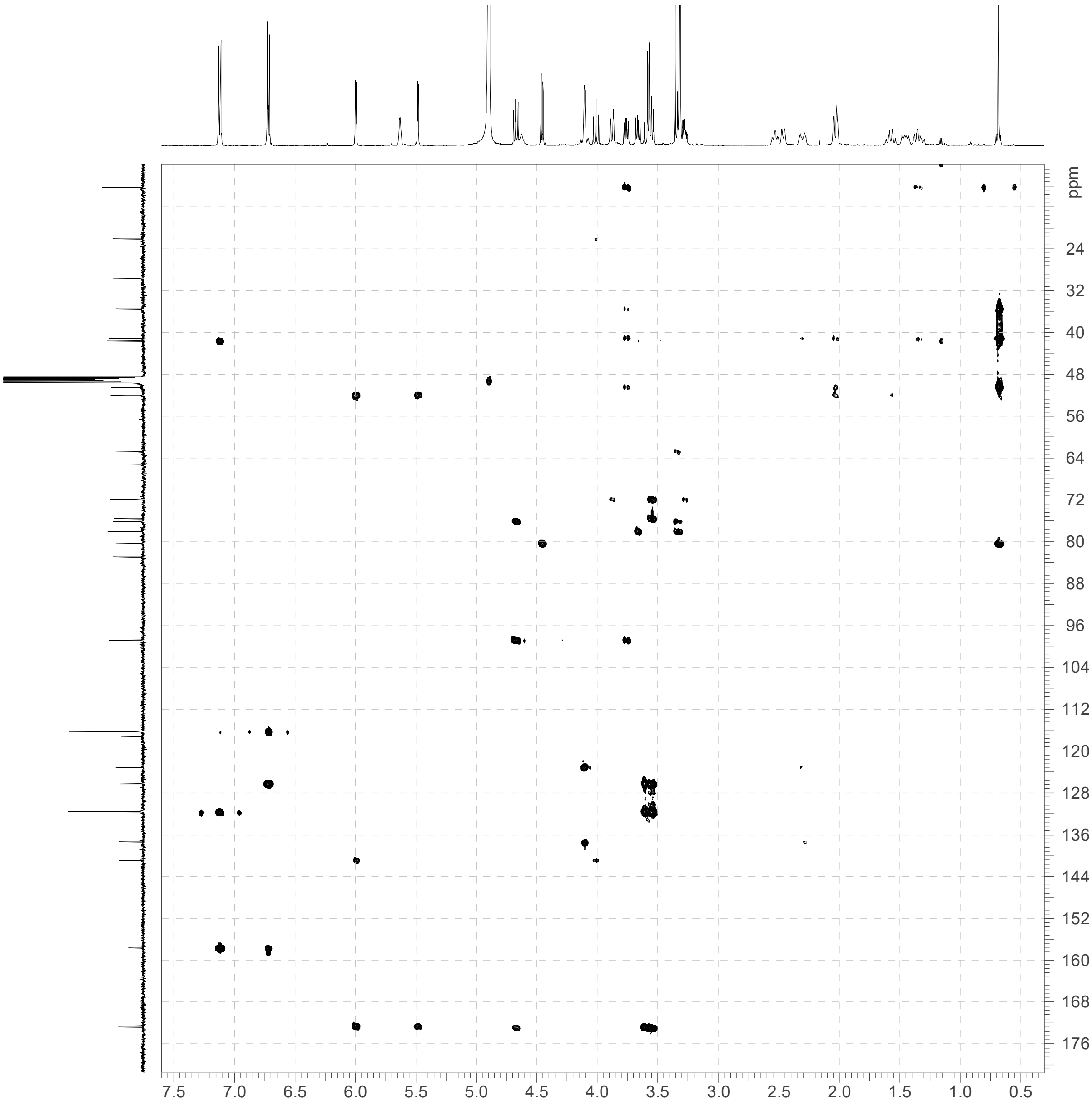

Figure S12. HMBC spectrum (500 MHz) of compound **4** in CD_3OD.

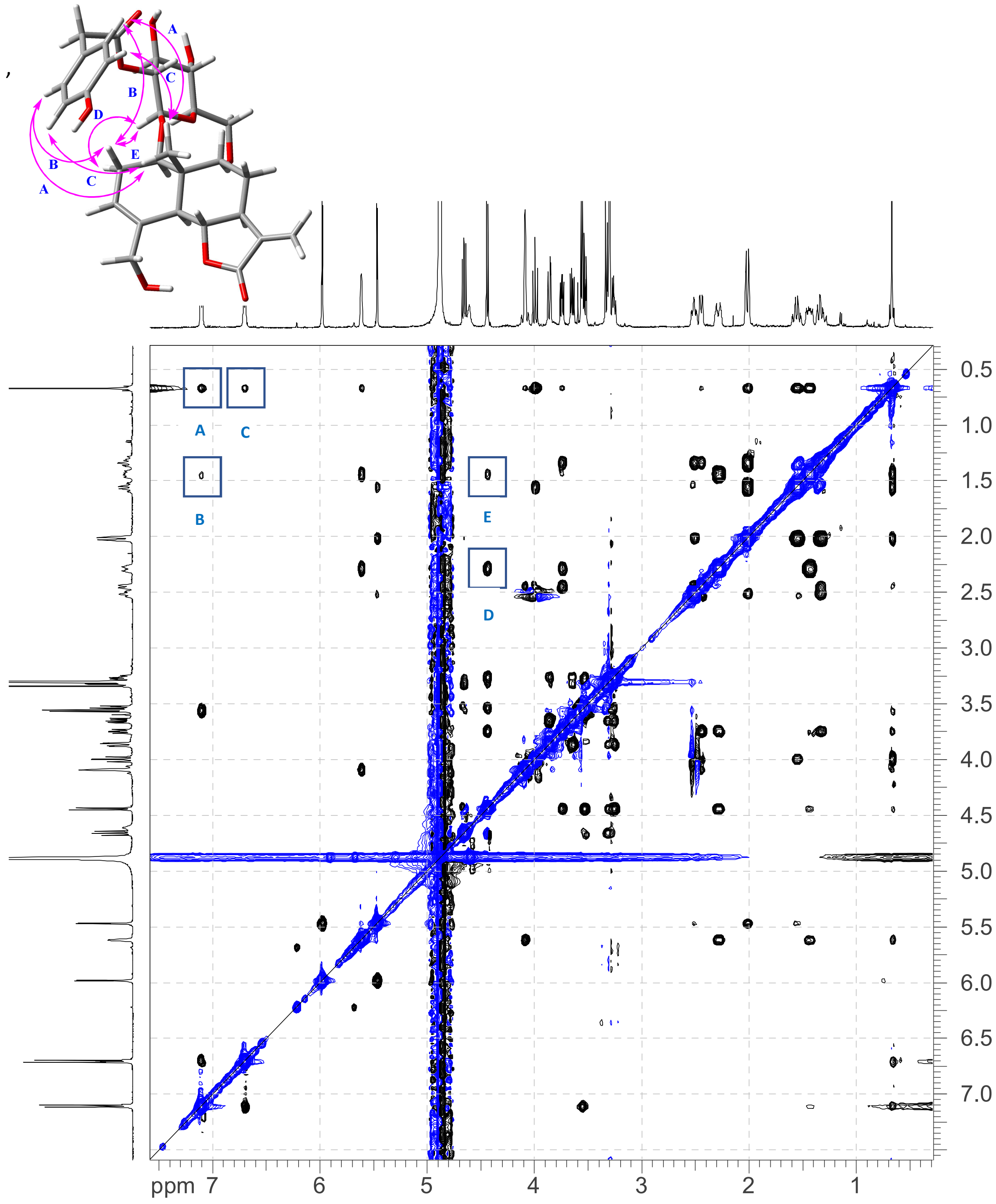

Figure S13. ROESY (500 MHz) spectrum of compound **4** in CD_3OD.

(A)

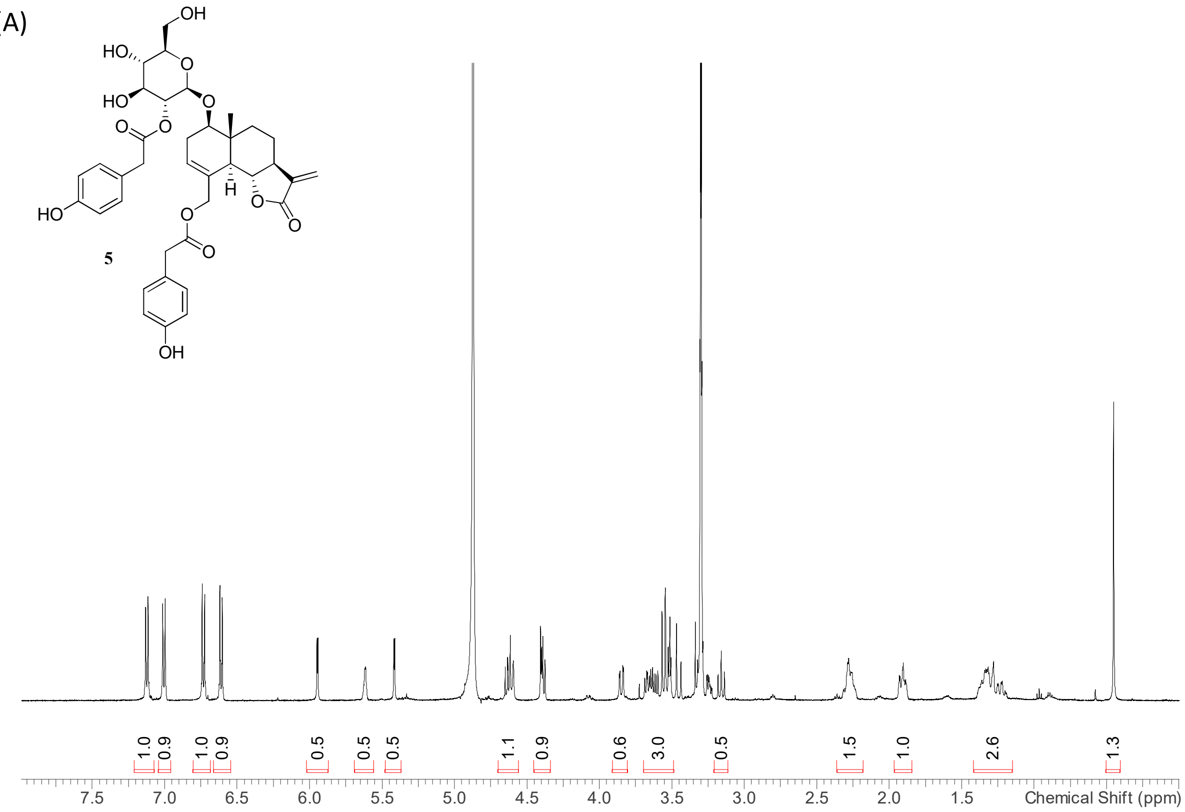

(B)

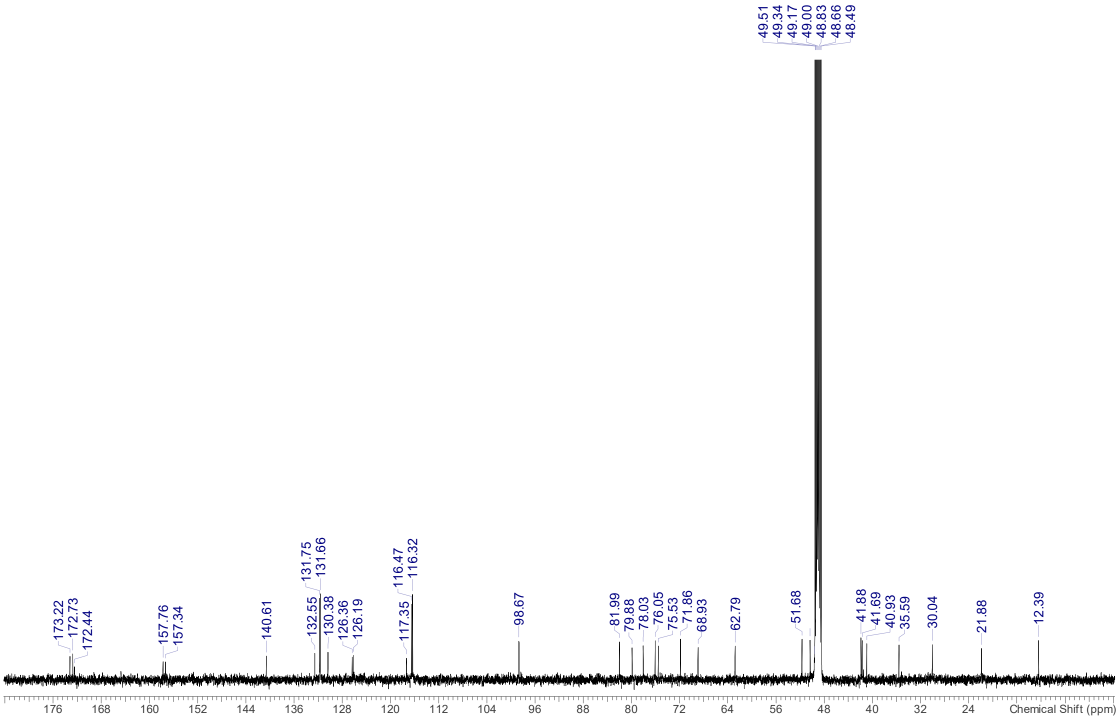

Figure S14. (A) ^{1}H (500 MHz) and (B) ^{13}C NMR (125 MHz) spectra of compound **5** In CD_3OD.

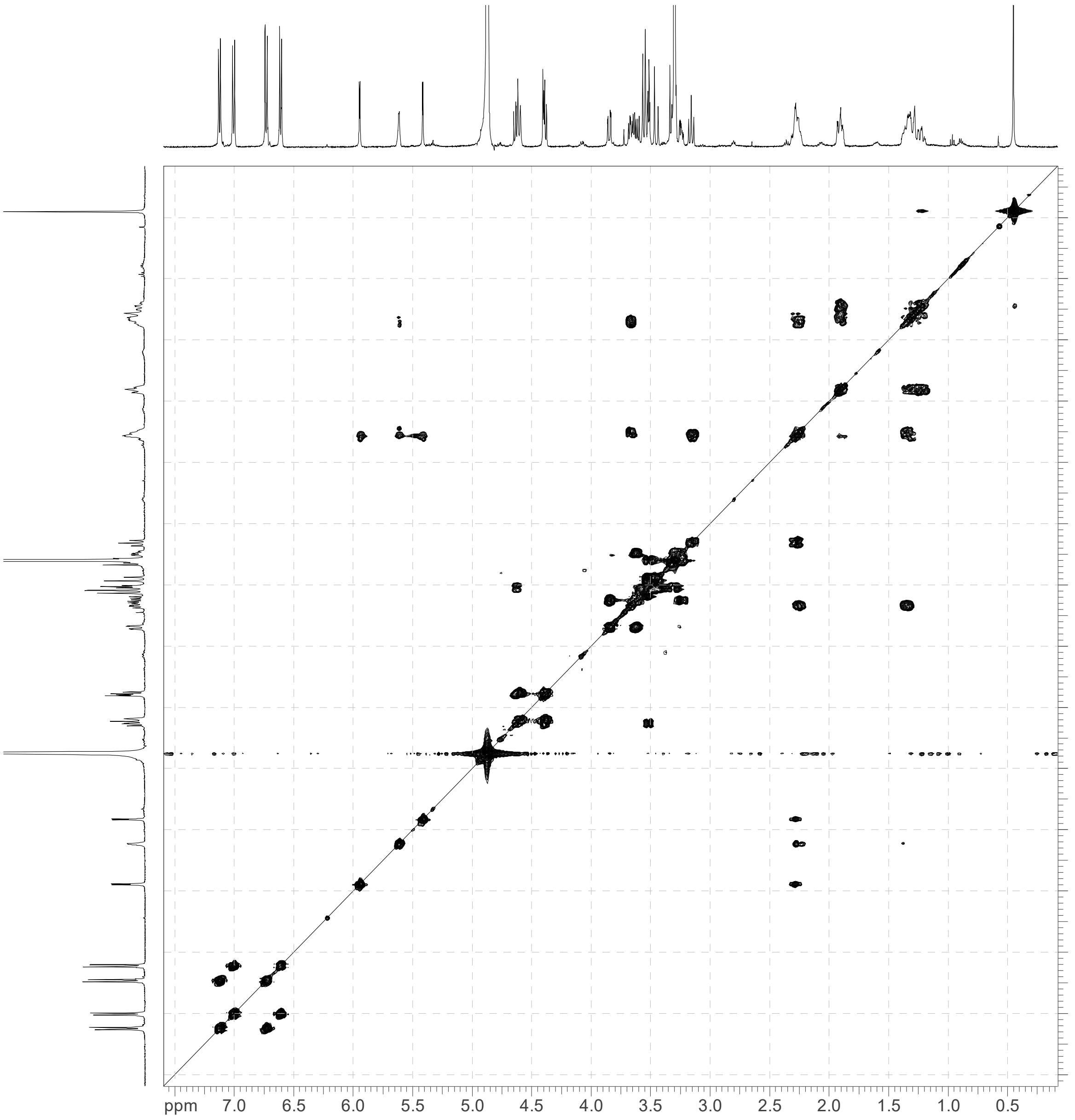

Figure S15. COSY spectrum (500 MHz) of compound **5** in CD_3OD.

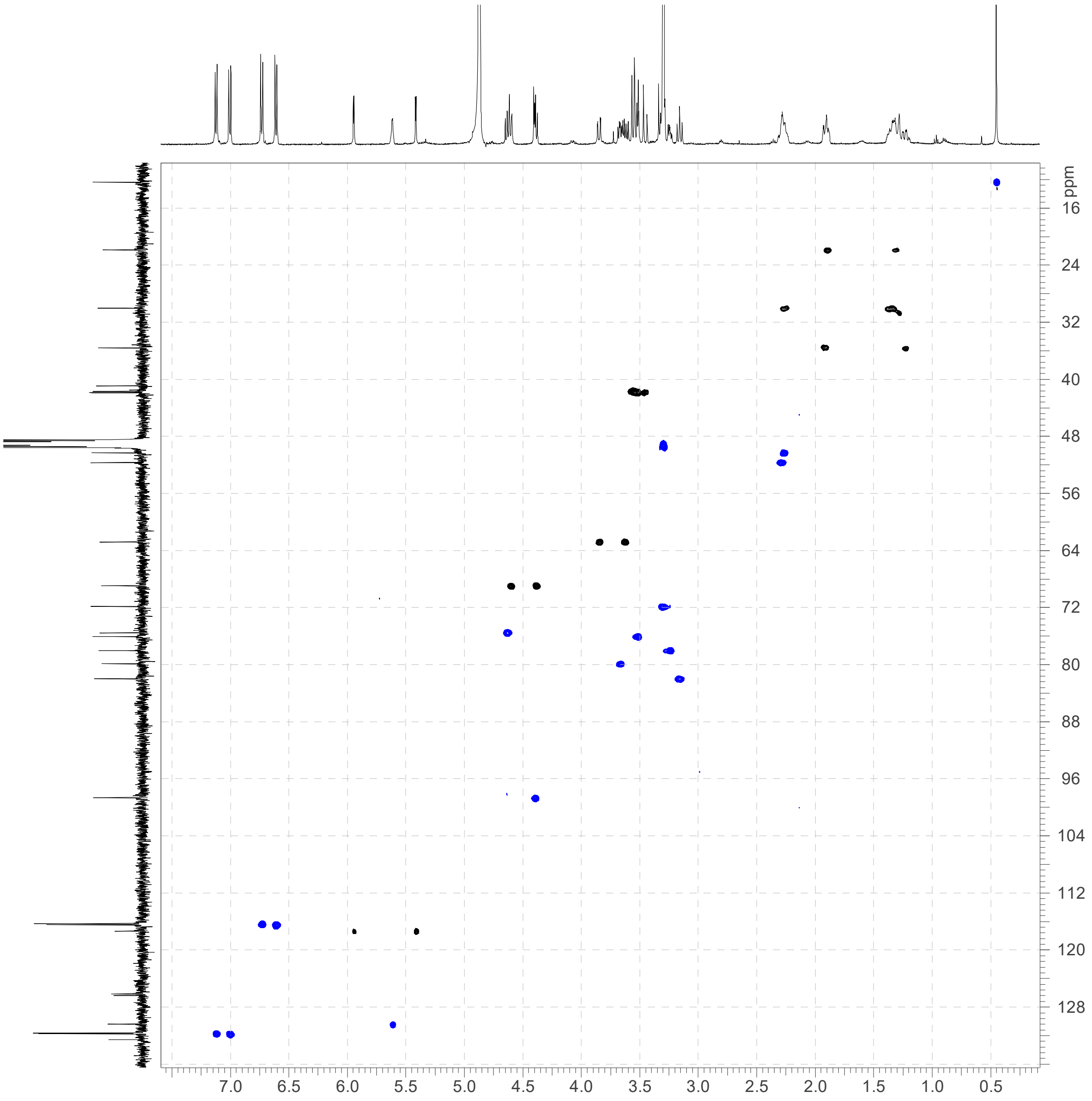

(blue: CH/CH$_3$; black: CH$_2$)

Figure S16. HSQC spectrum (500 MHz) of compound **5** in CD_3OD.

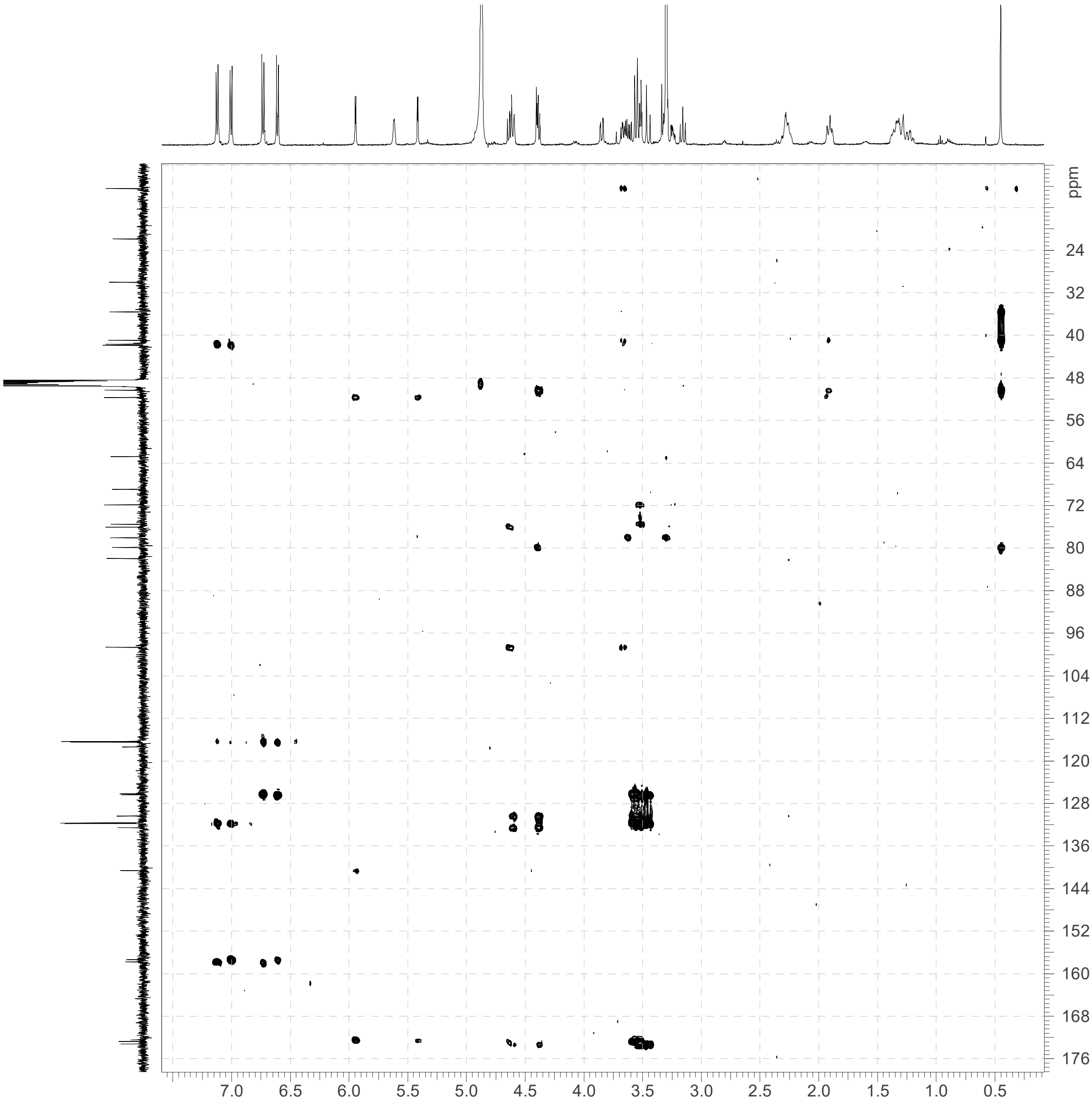

Figure S17. HMBC spectrum (500 MHz) of compound **5** in CD_3OD.

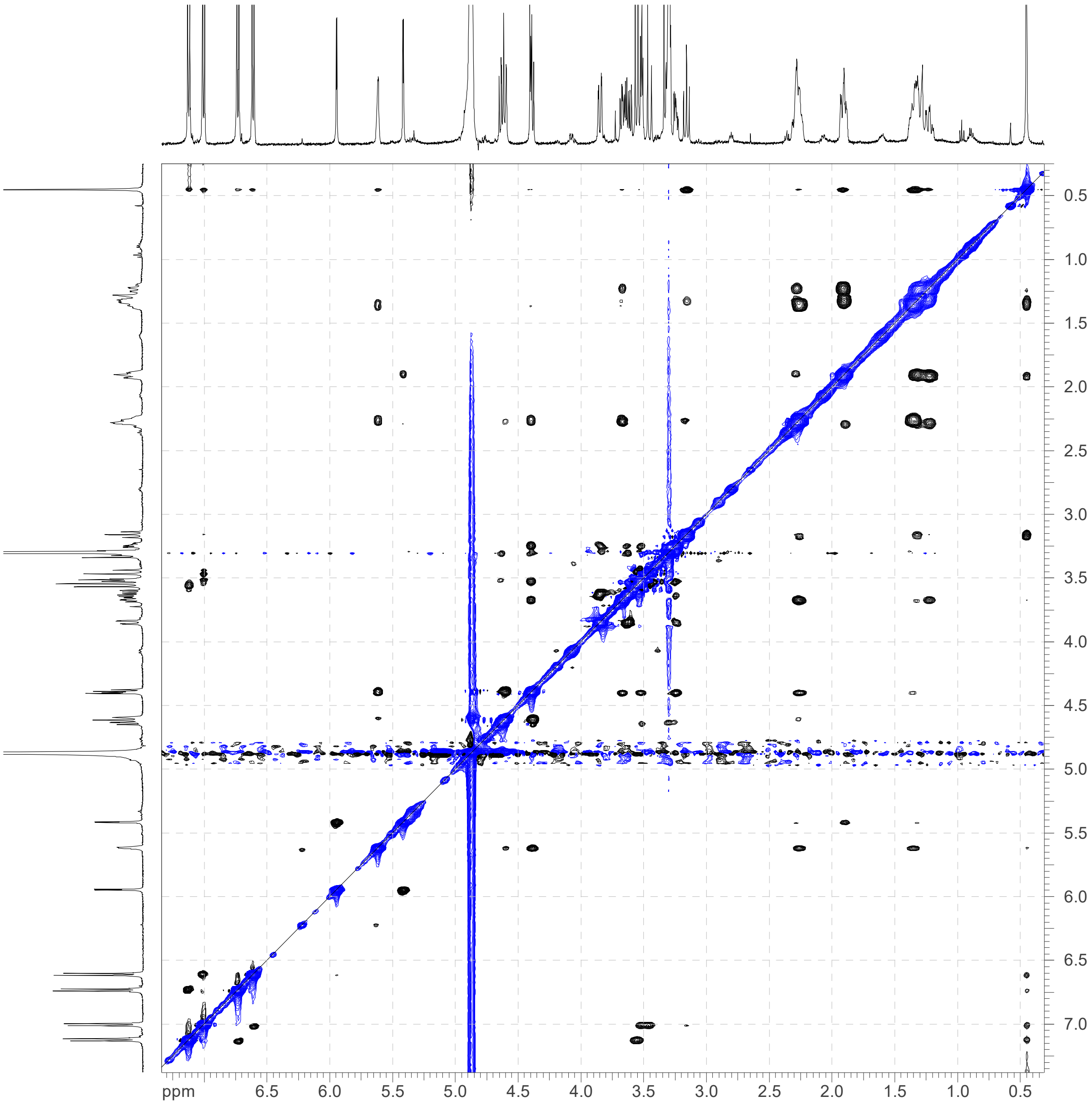

Figure S18. ROESY (500 MHz) spectrum of compound **5** in CD_3OD.
